# Cyclic Fatigue Resistance of Blue Heat-Treated Instruments at Different Temperatures

**DOI:** 10.1155/2021/5584766

**Published:** 2021-07-30

**Authors:** Thalita Miranda Vieira, Ryhan Menezes Cardoso, Nayane Chagas Carvalho Alves, Silvio Emanuel Acioly Conrado de Menezes, Shirley Machado Batista, Silmara de Andrade Silva, Christianne Velozo, Diana Santana de Albuquerque, Gabriela Queiroz de Melo Monteiro

**Affiliations:** Department of Operative Dentistry and Endodontics, Dental College of Pernambuco, University of Pernambuco (UPE), Camaragibe, PE, Zip Code 54756-220, Brazil

## Abstract

The main aim is to evaluate the cyclic fatigue resistance of blue heat-treated instruments with different kinematics. Twenty-four endodontic instruments of the same brand were used for each of three experimental groups: VB (Vortex Blue 40/0.04), RB (RECIPROC Blue 40/0.06), and XB (X1 Blue 40/0.06). The instruments were randomly distributed and subjected to temperatures of 20°C and 37°C. The fatigue test was performed using a stainless steel device. Data were analysed using the Shapiro–Wilk test, Student's *t-*test, the *F* test, and Tukey's and Tamhane tests at significance level *P*=0.05. The instruments' cyclic fatigue resistance at both temperatures differed significantly for each instrument type (*P* < 0.001). The RB instruments displayed greater cyclic fatigue resistance at the tested temperatures compared with the VB and XB instruments (*P* < 0.001). Reciprocating kinematics positively influenced cyclic fatigue resistance. Blue heat-treated instruments showed decreased cyclic fatigue resistance as the temperature increased (*P* < 0.001).

## 1. Introduction

Cyclic fatigue is one of the main mechanisms leading to nickel titanium (NiTi) instrument fracture in root canals [1–4]. This type of fatigue is caused by alternating tensile and compressive stresses when the instrument is inside a curved canal, and the instrument typically fractures in the area of maximum curvature [5, 6]. To overcome this limitation, instrument design and manufacturing methods have been improved, and treatments are applied to the NiTi alloy that provides superior mechanical properties [7–9].

These treatments are methods to transform shape memory properties and influence the mechanical behavior of the alloy, exhibiting better performance when comparing similar instruments made of conventional superelastic NiTi wires [10, 11]. The components' transition temperatures are adjusted to change the crystalline arrangement of the NiTi alloy atoms and modify it from the austenitic phase (cubic structure) to the martensitic phase (low-temperature phase, with a monoclinic B190 structure) [8]. A high percentage of this phase increases the instruments' flexibility and cyclic fatigue resistance [12–14] since the change to the martensitic phase has dampening characteristics, making crack propagation more difficult because more interfaces are present [15].

Automated instruments tested at different temperatures have considerably different fatigue behaviours [16, 17]. An increase to approximately 37°C (simulating body temperature) alters their fracture resistance [18–20], and since they are used within the canal, which is surrounded by the periodontium, body temperature is a relevant factor [21, 22].

Manufacturers have developed a special thermal process to increase the flexibility of NiTi alloy, which primarily contains a stable martensitic phase under clinical conditions [23, 24]. The blue thermal treatment imparts a blue colour and controls the temperature transition, ensuring alloy memory control and absence of elastic memory, and has been cited as a relevant factor in increased cyclic fatigue resistance [12, 14, 25].

Regarding motion kinematics, *in vitro* cyclic fatigue tests show that reciprocating instruments exhibit better fatigue resistance. However, the mean time to fracture is not directly proportional to the increase in the number of reciprocations required for a complete rotation (360°) probably because the resulting speeds are not directly proportional [26].

Per their manufacturers, when subjected to blue thermal treatment, the instruments Vortex Blue (VB; DENTSPLY Tulsa Dental, Tulsa, Oklahoma, USA), RECIPROC Blue (RB; VDW, Munich, Bayern, Germany), and X1 Blue (XB; MK Life, Porto Alegre, Rio Grande do Sul, Brazil) are indicated when better cyclic fatigue resistance is desired. Thus, the present study evaluated the cyclic fatigue resistance of instruments with blue thermal treatment and different kinematics, continuous, and reciprocal rotation (Vortex Blue, RECIPROC Blue, and X1 Blue) at 20°C (±0.5°C) and 37°C (±0.5°C) simulating room temperature and the temperature inside the root canal, respectively. Considering that body temperature (around 37°C) can significantly affect the transformation temperatures of the NiTi crystalline phase, the cyclic fatigue behavior of NiTi files.

The null hypotheses tested were the absence of significant differences in cyclic fatigue resistance between blue heat-treated instruments with different kinematics at 20°C (±0.5°C) and 37°C (±0.5°C).

## 2. Materials and Methods

Three brands of blue heat-treated NiTi endodontic instruments with different kinematics were tested. Twenty-four new endodontic instruments were used for each of the three experimental groups: VB (Vortex Blue 40/0.04), RB (RECIPROC Blue 40/0.06), and XB (X1 Blue 40/0.06), all with size ISO 40 and a length of 25 mm and commercially available in the country of origin of the study. Prior to use, all instruments were checked by a single operator under a stereomicroscope (SteREO Discovery V12, ZEISS, Germany) at 16x to qualitatively standardize the instruments in terms of lack of defect or deformation, such as distortions or coarse burrs on the cutting blades. No signs of change were detected, and no instrument was discarded. Twenty-four instruments of the same brand, all from the same production batch, were randomly distributed (http://www.random.org) into two groups (*n* = 12) and subjected to the temperatures of 20°C and 37°C. The endodontic instruments were used with a 6 : 1 reduction handpiece (Sirona Dental Systems GmbH, Bensheim, Germany) coupled to the VDW.Silver® RECIPROC® motor (VDW, Munich, Germany). The VB group instruments (Vortex Blue 40/0.04) were used at a speed of 500 rpm and a torque of 1.3 Nmm. In the RB (RECIPROC Blue 40/0.06) and XB (X1 Blue 40/0.06) groups, the instruments were used in the “RECIPROC All” mode, with the same kinematics.

**Figure 1:**
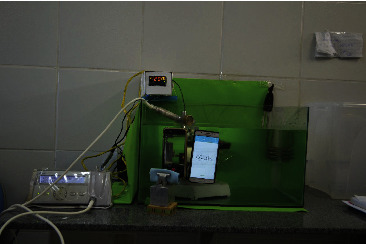
Custom device used for dynamic fatigue test.

The fatigue test was performed using a stainless steel device that allowed simulating instrument insertion into a curved artificial canal 1.5 mm wide [20]. A custom support (Figure 1) was used to keep the handpiece and steel canal static during use, allowing only free rotation of the instrument. The artificial canal was made with a 60° curvature angle and a 5 mm curvature radius measured per the Schneider [27] method and a centre of curvature at 5 mm from the end of the instrument. The instrument to be tested was coupled to the handpiece and inserted into the artificial canal parallel to the vertical portion of the canal, following the direction from the tip of the instrument to the base. The device was completely submerged in distilled water in a glass container measuring 50 × 20 × 30 cm [18, 20]. The temperature was controlled at 20°C (±0.5°C) or 37°C (±0.5°C) while working using a digital underwater thermometer and a thermostat [20]. To heat and cool the water during testing, a heating element and ice were used, respectively. The thermostat tip was sufficiently maintained near the simulated canal to ensure the specific temperature during analysis. All endodontic instruments were used per the manufacturers' recommendations and operated freely and statically in the axial direction until fracture. Fatigue resistance was determined by measuring the time in seconds via video recording, starting when the instrument was triggered and stopping when the fracture was visually detected [12]. The number of cycles to fracture (NCF) was calculated using the formula NCF = rotations per minute (rpm) × time to failure in seconds (s)/60 s. After fracture, the fragment length was measured using a digital caliper with 0.01 mm accuracy.

The data were descriptively analysed using SPSS 23.0 (SPSS Inc, Chicago, IL, USA) for Windows and Excel 2010. The normality of the data for each file type and temperature combination was tested using the Shapiro–Wilk test. The analysis was inferential, using Student's *t*-test with equal variances, Student's *t*-test with unequal variances and the *F* (analysis of variance (ANOVA)) test with Tukey's or Tamhane posttests. Tukey's multiple comparisons test was used in cases where the equality of variances hypothesis was confirmed, and the Tamhane comparisons test was used when the hypothesis of equality of variances was rejected. The significance level was set at *P*=0.05.

## 3. Results

**Figure 2:**
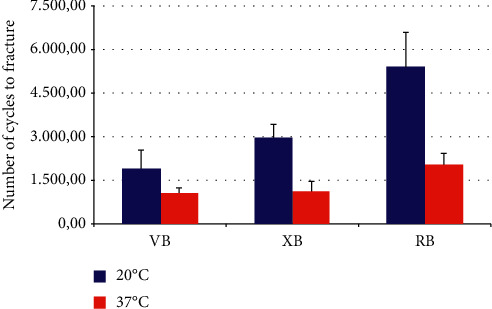
Mean and standard deviation of the number of cycles to fracture (NCF) according to the instrument type and temperature used.

The mean and standard deviation of the NCF values are shown in Table 1 and in Figure 2. The instruments' cyclic fatigue resistance at both temperatures (20°C and 37°C) differed significantly by the instrument type (*P* < 0.001). The RB instruments displayed greater cyclic fatigue resistance at the tested temperatures compared with the VB and XB instruments (*P* < 0.001). The multiple comparison tests showed that NCF significantly differed among the three instrument types at 20°C (*P*=0.004) and between the RB instruments and the other two brands at 37°C (*P* < 0.001). The mean fragment length (4.62–5.40 mm) did not differ significantly among the instruments tested at 20°C or 37°C (*P* > 0.05).

## 4. Discussion

The results of the present study showed that decreased temperatures increased the cyclic fatigue resistance of blue heat-treated instruments. These instruments are the result of the oxidation that heating and cooling induce on their surface, making the NiTi alloy more martensite, hence giving it better fatigue life [18]. To date, no cyclic fatigue studies have compared only blue heat-treated instruments (VB, RB, and XB) under different temperatures and kinematics. Temperature has been investigated as a variable that considerably influences cyclic fatigue resistance [16, 18, 19]. Increased temperature causes decreased cyclic fatigue resistance [17, 20, 22, 28], as our findings showed. At 20°C, the NCF values for each instrument type were significantly higher than those at 37°C (*P* < 0.001), for groups XB and RB. The NCF values among the three instrument types tested differed significantly at 20°C (*P*=0.004). At 37°C, this difference occurred for the RB instruments relative to the VB and XB instruments (*P* < 0.001). Thus, the null hypotheses were rejected (*P* < 0.05).

In the past, most cyclic fatigue resistance studies were performed at room temperature, but this did not reflect clinical conditions, as the root canal had a different temperature [29]. The environmental conditions in which the cyclic fatigue test is performed affect the fracture resistance as well as motion kinematics, metal alloy, and physical properties of the instruments [16, 30, 31]. According to our results, the ideal temperature to verify the resistance to cyclic fatigue was 37°C, simulating the root canal temperature. For this, we use a thermostat keeping the temperature constant.

An ideal experimental model for testing cyclic fatigue would involve instrumentation in natural teeth. However, sample standardization is difficult and restricts the sample size because of the large variation in length, radius, and degree of curvature within the same dental group [32]. In addition, testing all instruments on the same tooth is impossible because the sample is permanently changed after instrumentation. Another important variable is performance of the cyclic fatigue test in air, using oil or immersed in water, which may influence the NCF values. Therefore, the tests were conducted in artificial canals immersed in water to ensure that the experimental conditions were standardized and with great temperature control as in previous studies [17–20, 28]. Notably, no specific ISO standard exists for validating a device to perform cyclic fatigue tests [28]; thus, a device was created for this study.

Reciprocating motion kinematics extend the useful life of the instruments because they expose the instruments to lower voltage values compared to continuous rotation, thus increasing NCF [33–35]. Through a systematic review, Ferreira et al. [26] noted that the reciprocating motion improves the instruments' cyclic fatigue resistance compared with continuous rotation independent of other variables, such as the rotational velocity, curvature angle of the simulated canal, and NiTi instrument design. This information agrees with the results of this study, as the reciprocating RB and XB instruments presented higher NCF compared with the rotating VB instrument, although the latter had the lowest taper (0.04).

A smaller radius of curvature and an increased instrument diameter result in fewer cycles to fracture [36]. The instruments tested in our study had an elevated diameter—40—and were thus more susceptible to fatigue than instruments with smaller diameters [36, 37]. However, the RB and XB instruments had the same taper of 0.06, while that of the VB instrument was 0.04. The RB instruments exhibited significantly higher NCF than did the XB instruments, which had the same taper, and the VB instruments, which had a lower taper. This finding shows that, in blue heat-treated instruments, the variable taper did not appear to affect cyclic fatigue resistance. Additionally, the RB instruments had an S-shaped cross section, unlike the triangular cross section present in the other two instruments tested. This finding corroborates the findings of Kaval et al. [38], who found a higher NCF in instruments with S-shaped cross-sections, which have a smaller metal core than those with triangular cross-sections.

The blue heat-treated XB reciprocating instruments, produced with controlled memory (CM) NiTi alloy, were recently launched on the market. They have an inactive tip and a triangular cross section per the manufacturer's instryctions. No studies have reported XB file behaviour (40/0.06) in cyclic fatigue tests. In our study, the NCFs of these instruments at 20°C were significantly smaller and larger, respectively, than those of the RB and VB instruments (*P* < 0.05). At 37°C, the XB instruments differed only from the RB instruments (*P* < 0.001), presenting a lower NCF. When compared between the temperatures tested, the NCFs of the XB instruments differed significantly.

The VB rotary instruments manufactured with CM NiTi alloy and blue heat treatment are characterized by a constant taper, a triangular cross section, and the absence of radial lands [39, 40]. In our study, the VB instruments presented lower NCF than the other instruments at both tested temperatures, with values near those found by Dosanjh et al. [20] at the same angle and radius of curvature, showing a mean NCF of 1,233 at 37°C.

Some studies have suggested that cooling down to low temperatures may be an interesting strategy to improve the fatigue resistance of rotary NiTi files although cooling down to a low temperature during the instrumentation may be difficult to achieve [17]. This experiment was pioneering in evaluating the cyclic fatigue resistance of RB 40/0.06 instruments in canals with a 60° curvature angle and a 5 mm radius. Because of this condition, the NCF results for the RB 25/0.08 instruments are discussed. Studies with the same artificial canal condition showed that RB instruments (25/0.08) presented better cyclic fatigue resistance compared with the RECIPROC (25/0.08), WaveOne Gold Primary (25/0.07) [17, 34], and One Shape (25/0.06) [41] instruments, but none reported the experimental temperature. Consistent with these studies, even with higher-diameter instruments, our study found higher NCF values for RB 40/0.06. Thus, the null hypotheses were rejected.

## 5. Conclusion

Within the limitation of the study, the RECIPROC Blue instruments showed greater cyclic fatigue resistance compared with the X1 Blue and Vortex Blue instruments at 20°C and 37°C. The results indicate that the fatigue test should be conducted at body temperature instead of room temperature.

## Figures and Tables

**Table 1:** Mean and standard deviation of the number of cycles to fracture (NCF) according to instrument type and temperature used.

Instrument type	20°C mean ± SD (CV%)	37°C mean ± SD (CV%)	*P* value
VB	1.899 ± 629.73^(A)^	1.033 ± 190.07^(A)^	*P* ^(1)^ < 0.001^*∗*^
XB	2.974 ± 449.12^(B)^	1.082 ± 374.19^(A)^	*P* ^(2)^ < 0.001^*∗*^
RB	5.419 ± 1,179.85^(C)^	2.019 ± 388.49^(B)^	*P* ^(1)^ < 0.001^*∗*^
*P* value	**P** ^**(3)**^ **=** **0.004**^*∗*^	**P** ^**(4)**^ **<** **0.001**^*∗*^	

^*∗*^Significant difference at 0.05. ^(1)^Student's *t-*test with equal variances. ^(2)^Student's *t-*test with unequal variances. ^(3)^*F* (analysis of variance (ANOVA)) test with Tukey's comparisons. ^(4)^*F* (ANOVA) test with Tamhane comparisons. Different superscript letters in the same column mean significant difference (*P* < 0.01).

## Data Availability

The data used to support the findings of this study are included within the article.

## References

[1] Parashos P., Gordon I., Messer H. (2004). Factors influencing defects of rotary nickel-titanium endodontic instruments after clinical use. *Journal of Endodontics*.

[2] Peng B., Shen Y., Cheung G. S. P., Xia T. J. (2005). Defects in protaper S1 instruments after clinical use: longitudinal examination. *International Endodontic Journal*.

[3] Robertson S. W., Pelton A. R., Ritchie R. O. (2012). Mechanical fatigue and fracture of nitinol. *International Materials Reviews*.

[4] Shen Y., Zhou H., Campbell L. (2014). Fatigue and nanomechanical properties of K3XF nickel-titanium instruments. *International Endodontic Journal*.

[5] Pedullà E., Grande N. M., Plotino G., Gambarini G., Rapisarda E. (2013). Influence of continuous or reciprocating motion on cyclic fatigue resistance of 4 different nickel-titanium rotary instruments. *Journal of Endodontics*.

[6] Shen Y., Cheung G. S.-p., Bian Z., Peng B. (2006). Comparison of defects in profile and protaper systems after clinical use. *Journal of Endodontics*.

[7] Gambarini G., Grande N. M., Plotino G. (2008). Fatigue resistance of engine-driven rotary nickel-titanium instruments produced by new manufacturing methods. *Journal of Endodontics*.

[8] Shen Y., Zhou H.-m., Zheng Y.-f., Peng B., Haapasalo M. (2013). Current challenges and concepts of the thermomechanical treatment of nickel-titanium instruments. *Journal of Endodontics*.

[9] Yahata Y., Yoneyama T., Hayashi Y. (2009). Effect of heat treatment on transformation temperatures and bending properties of nickel-titanium endodontic instruments. *International Endodontic Journal*.

[10] Ye J., Gao Y. (2012). Metallurgical characterization of M-wire nickel-titanium shape memory alloy used for endodontic rotary instruments during low-cycle fatigue. *Journal of Endodontics*.

[11] Zhou H.-m., Shen Y., Zheng W., Li L., Zheng Y.-f., Haapasalo M. (2012). Mechanical properties of controlled memory and superelastic nickel-titanium wires used in the manufacture of rotary endodontic instruments. *Journal of Endodontics*.

[12] De-Deus G., Silva E. J. N. L., Vieira V. T. L. (2017). Blue thermomechanical treatment optimizes fatigue resistance and flexibility of the reciproc files. *Journal of Endodontics*.

[13] Duke F., Shen Y., Zhou H. (2015). Cyclic fatigue of profile vortex and vortex blue nickel-titanium files in single and double curvatures. *Journal of Endodontics*.

[14] Plotino G., Grande N. M., Cotti E., Testarelli L., Gambarini G. (2014). Blue treatment enhances cyclic fatigue resistance of vortex nickel-titanium rotary files. *Journal of Endodontics*.

[15] Pereira E. S. J., Gomes R. O., Leroy A. M. F. (2013). Mechanical behavior of M-wire and conventional NiTi wire used to manufacture rotary endodontic instruments. *Dental Materials*.

[16] Grande N. M., Plotino G., Silla E. (2017). Environmental temperature drastically affects flexural fatigue resistance of nickel-titanium rotary files. *Journal of Endodontics*.

[17] Shen Y., Huang X., Wang Z., Wei X., Haapasalo M. (2018). Low environmental temperature influences the fatigue resistance of nickel-titanium files. *Journal of Endodontics*.

[18] Arias A., Macorra J. C., Govindjee S., Peters O. A. (2018). Correlation between temperature-dependent fatigue resistance and differential scanning calorimetry analysis for 2 contemporary rotary instruments. *Journal of Endodontics*.

[19] De Vasconcelos R. A., Murphy S., Carvalho C. A. T., Govindjee R. G., Govindjee S., Peters O. A. (2016). Evidence for reduced fatigue resistance of contemporary rotary instruments exposed to body temperature. *Journal of Endodontics*.

[20] Dosanjh A., Paurazas S., Askar M. (2017). The effect of temperature on cyclic fatigue of nickel-titanium rotary endodontic instruments. *Journal of Endodontics*.

[21] De Hemptinne F., Slaus G., Vandendael M., Jacquet W., De Moor R. J., Bottenberg P. (2015). In vivo intracanal temperature evolution during endodontic treatment after the injection of room temperature or preheated sodium hypochlorite. *Journal of Endodontics*.

[22] Jamleh A., Kobayashi C., Yahata Y., Ebihara A., Suda H. (2012). Deflecting load of nickel titanium rotary instruments during cyclic fatigue. *Dental Materials Journal*.

[23] Pereira E. S. J., Peixoto I. F. C., Viana A. C. D. (2012). Physical and mechanical properties of a thermomechanically treated NiTi wire used in the manufacture of rotary endodontic instruments. *International Endodontic Journal*.

[24] Wayman C. M., Duerig T. W., Duerig T. W., Melton K. N., Stöckel D., Wayman C. M. (1990). An introduction to martensite and shape memory. *Engineering Aspects of Shape Memory Alloys*.

[25] Gao Y., Gutmann J. L., Wilkinson K., Maxwell R., Ammon D. (2012). Evaluation of the impact of raw materials on the fatigue and mechanical properties of profile vortex rotary instruments. *Journal of Endodontics*.

[26] Ferreira F., Adeodato C., Barbosa I., Aboud L., Scelza P., Zaccaro Scelza M. (2017). Movement kinematics and cyclic fatigue of NiTi rotary instruments: a systematic review. *International Endodontic Journal*.

[27] Schneider S. W. (1971). A comparison of canal preparations in straight and curved root canals. *Oral Surgery, Oral Medicine, Oral Pathology*.

[28] Plotino G., Grande N. M., Cordaro M., Testarelli L., Gambarini G. (2009). A review of cyclic fatigue testing of nickel-titanium rotary instruments. *Journal of Endodontics*.

[29] Uygun A. D. (2020). Cyclic fatigue resistance of VDW rotate and reciproc blue nickel-titanium files at root canal temperature. *Journal of Dental Research, Dental Clinics, Dental Prospects*.

[30] Plotino G., Grande N. M., Mercadé Bellido M., Testarelli L., Gambarini G. (2017). Influence of temperature on cyclic fatigue resistance of protaper gold and protaper universal rotary files. *Journal of Endodontics*.

[31] Klymus M. E., Alcalde M. P., Vivan R. R., Só M. V. R., de Vasconselos B. C., Duarte M. A. H. (2019). Effect of temperature on the cyclic fatigue resistance of thermally treated reciprocating instruments. *Clinical Oral Investigations*.

[32] Santa-Rosa J., De Sousa-Neto M. D., Versiani M. A. (2016). Shaping ability of single-file systems with different movements: a micro-computed tomographic study. *Iranian Endodontic Journal*.

[33] Ahn S.-Y., Kim H.-C., Kim E. (2016). Kinematic effects of nickel-titanium instruments with reciprocating or continuous rotation motion: a systematic review of in vitro studies. *Journal of Endodontics*.

[34] Keskin C., Inan U., Demiral M., Keleş A. (2017). Cyclic fatigue resistance of reciproc blue, reciproc, and waveone gold reciprocating instruments. *Journal of Endodontics*.

[35] Tokita D., Ebihara A., Miyara K., Okiji T. (2017). Dynamic torsional and cyclic fracture behavior of profile rotary instruments at continuous or reciprocating rotation as visualized with high-speed digital video imaging. *Journal of Endodontics*.

[36] Pruett J. P., Clement D. J., Carnes D. L. (1997). Cyclic fatigue testing of nickel-titanium endodontic instruments. *Journal of Endodontics*.

[37] Arias A., Hejlawy S., Murphy S., de la Macorra J. C., Govindjee S., Peters O. A. (2019). Variable impact by ambient temperature on fatigue resistance of heat-treated nickel titanium instruments. *Clinical Oral Investigations*.

[38] Kaval M. E., Capar I. D., Ertas H., Sen B. H. (2017). Comparative evaluation of cyclic fatigue resistance of four different nickel-titanium rotary files with different cross-sectional designs and alloy properties. *Clinical Oral Investigations*.

[39] Gutmann J. L., Gao Y. (2012). Alteration in the inherent metallic and surface properties of nickel-titanium root canal instruments to enhance performance, durability and safety: a focused review. *International Endodontic Journal*.

[40] Nguyen H. H., Fong H., Paranjpe A., Flake N. M., Johnson J. D., Peters O. A. (2014). Evaluation of the resistance to cyclic fatigue among protaper next, protaper universal, and vortex blue rotary instruments. *Journal of Endodontics*.

[41] Gündoğar M., Özyürek T. (2017). Cyclic fatigue resistance of oneshape, hyflex EDM, waveone gold, and reciproc blue nickel-titanium instruments. *Journal of Endodontics*.

